# [^131^I]6ß-Iodomethyl-19-norcholesterol SPECT/CT for the Lateralization of Mineralocorticoid Overproduction in Primary Aldosteronism

**DOI:** 10.3390/diagnostics14171997

**Published:** 2024-09-09

**Authors:** Sandor Barna, Livia Sira, Harjit Pal Bhattoa, Laszlo Toth, Zsigmond Czine, Lilla Szoboszlay, Edit B. Nagy, Zita Kepes, Ildiko Garai, Miklos Bodor, Jozsef Varga, Endre V. Nagy

**Affiliations:** 1 Scanomed Nuclear Medicine Center, 98 Nagyerdei krt, 4032 Debrecen, Hungary; barna.sandor@scanomed.hu (S.B.); garai.ildiko@med.unideb.hu (I.G.); 2 Division of Nuclear Medicine and Translational Imaging, Department of Medical Imaging, Faculty of Medicine, University of Debrecen, 4032 Debrecen, Hungary; kepes.zita@med.unideb.hu (Z.K.); jvarga@med.unideb.hu (J.V.); 3 Division of Endocrinology, Department of Internal Medicine, Faculty of Medicine, University of Debrecen, 4032 Debrecen, Hungary; sira.livia@belklinika.com (L.S.); mbodor@gmail.com (M.B.); 4 Department of Laboratory Medicine, Faculty of Medicine, University of Debrecen, 4032 Debrecen, Hungary; harjit@med.unideb.hu; 5 Department of Pathology, Faculty of Medicine, University of Debrecen, 4032 Debrecen, Hungary; tothlasz@med.unideb.hu; 6 Department of Internal Medicine, Jósa András Szabolcs Szatmár Bereg County Teaching Hospital, 4246 Nyiregyhaza, Hungary; zsigaman@yahoo.com; 7 Health Care Service Units, Department of Internal Medicine, Gróf Tisza Istvan Campus, University of Debrecen, 4032 Debrecen, Hungary; szoboszlay88@gmail.com; 8 Division of Radiology, Department of Medical Imaging, Faculty of Medicine, University of Debrecen, 4032 Debrecen, Hungary; nagy.edit@med.unideb.hu

**Keywords:** hypertension, primary aldosteronism, [^131^I]6ß-iodomethyl-19-norcholesterol, SPECT/CT

## Abstract

Primary: aldosteronism is a frequent cause of secondary hypertension. With access to specialized care, an increasing number of patients with aldosteronism are being identified. Primary aldosteronism is treatable by adrenal surgery if aldosterone excess originates from one of the two, and not from both, adrenals. Bilateral hyperplasia requires lifelong mineralocorticoid receptor antagonist treatment. Up till now, adrenal venous sampling (AVS) has been widely used to distinguish between one-sided and two-sided aldosterone overproduction and patient selection for surgery. AVS is an invasive technique, and the unsuccessful sampling of the right adrenal vein during AVS often prevents side comparison, making the AVS procedure useless. Molecular imaging using [^131^I]6ß-iodomethyl-19-norcholesterol with SPECT CT imaging (SPECT/CT) may be a potential alternative. Methods: In 42 consecutive patients with confirmed primary aldosteronism, molecular imaging has been performed. After dexamethasone suppression of the non-affected adrenal tissue, 37 MBq [^131^I]6ß-iodomethyl-19-norcholesterol was injected i.v., and SPECT/CT images were taken 7 days later. Based on the visual evaluation of the images by two nuclear medicine specialists, patients with one-sided tracer accumulation underwent adrenalectomy. To identify a SPECT/CT parameter that best characterizes the side difference, the maximum counts and the mean counts of spherical VOIs were analyzed. Results: Of the 42 patients, 24 had one-sided aldosterone overproduction by SPECT/CT. After surgical removal of the involved adrenal, all 24 patients with SPECT/CT-identified unilateral aldosteronism achieved biochemical cure, defined as a normalized potassium level combined with an aldosterone-to-renin ratio ≤ 30. To identify the best measurable parameter of SPECT/CT side difference, the mean counts and maximum counts of a series of spherical VOIs of different diameters were analyzed. The ratio of the mean counts of 3 cm spherical VOIs of the right and left adrenal regions (lateralization index) was the best discriminator; a ratio of ≥1.29 was characteristic of one-sided disease, without overlap between the one-sided and two-sided patient groups. Conclusions: [^131^I]6ß-iodomethyl-19-norcholesterol SPECT/CT with a count-based image interpretation and side-ratio calculation may be an equipollent non-invasive substitute for adrenal venous sampling in the lateralization of mineralocorticoid overproduction. It reliably identifies unilateral disease and facilitates patients’ selection for surgical intervention. If confirmed by others, this functional imaging may replace AVS when lateralization is required for management decisions in primary aldosteronism.

## 1. Introduction

Primary aldosteronism (PA) is a frequent and often unrecognized condition that may account for or contribute to high blood pressure in 12 to 22% of patients, the prevalence depending on the severity of hypertension [1]. PA-related elevated blood pressure is associated with higher cardiovascular morbidity compared to primary hypertension [2]. Early identification and treatment of PA is mandatory to avoid life-threatening PA-linked consequences [3].

Once PA has been proven, or to put it in a broader view, once PA could not be excluded by screening in a patient with hypokalemia and/or resistant hypertension, one of the central issues of the workup is lateralization of aldosterone overproduction. Until recently, the two major causes of primary aldosteronism had been aldosterone-producing adenoma (APA) and bilateral hyperaldosteronism (BHA). Although recent findings point towards a more multifaceted spectrum disease, the distinction between predominantly unilateral or bilateral disease remains crucial, because for unilateral disease, surgery may result in a complete cure in young patients with a short history of hypertension and may allow for a substantial reduction in the number of antihypertensive drugs in others [4]. For bilateral disease, lifelong medical treatment is warranted. Conventional imaging, such as computed tomography (CT) or magnetic resonance imaging (MRI), can detect adrenal pathologies; however, these modalities provide no information on hormone production of the detected one-sided lesion due to the frequent dichotomy of CT morphology and aldosterone secretion of the given adrenal [5,6,7]. Except for patients younger than 35 years with spontaneous hypokalemia and marked aldosterone excess where CT may be sufficient, adrenal venous sampling (AVS) is the primary recommended diagnostic modality for the differentiation between unilateral and bilateral disease [8,9]. AVS provides a reliable side comparison when venous sampling is successful. However, the sampling of the right adrenal vein may be challenging. The best published overall AVS success rate in a high-volume center was 92% [10], exceeding the 80% reported success in a major study of 1625 patients [11]. The AVS procedure itself is considered invasive, and when offered, a substantial number of patients decline the catheterization and choose mineralocorticoid receptor antagonist therapy instead after shared decision making. Therefore, there is room for the establishment of sensitive, less invasive modalities for the lateralization of aldosterone overproduction.

The idea of functional imaging in PA is not new. Planar gamma camera adrenal imaging with [^131^I]6ß-iodomethyl-19-norcholesterol was tried as early as 48 years ago [12]. Here, we evaluate [^131^I]6ß-iodomethyl-19-norcholesterol adrenal SPECT/CT (ICAS) using the surgical outcome as the measure of diagnostic performance in patients with PA. We show that ICAS can reliably lateralize aldosterone secretion and may be a non-invasive substitute for AVS.

## 2. Materials and Methods

### 2.1. Study Participants

Between 2017 and 2023, forty-two consecutive patients with confirmed PA were enrolled in the study. Patients unlikely to accept adrenalectomy if recommended were not considered for inclusion. Clinical data, including medication history, serum aldosterone level, plasma renin activity (PRA), aldosterone-to-renin ratio (ARR), and potassium levels, of each patient were reviewed by two endocrinologists. ARR > 30 off interfering medications was required to be considered for enrollment. PA was then confirmed by either the simultaneous presence of spontaneous low serum potassium and plasma aldosterone > 550 pmol/L and PRA below the level of detection, or a plasma aldosterone > 190 pmol/L after 2 L iv. 0.9% saline loading. This protocol followed the Endocrine Society Guideline [8], except that when AVS was required, ICAS was performed instead.

All followed procedures were in accordance with the ethical standards of the Declaration of Helsinki. The study was approved by the Regional and Institutional Ethics Committee of the University of Debrecen (5910-2016/DE RKEB/IKEB). Before enrolment, subjects were given detailed information regarding both the aims of the study and details of the examination. Informed consent was obtained from each patient.

### 2.2. Patient Preparation

With the aim of prohibiting [^131^I]6ß-iodomethyl-19-norcholesterol accumulation in the non-affected, normally functioning adrenal tissue, adrenocorticotropic hormone (ACTH) suppression was accomplished by administering dexamethasone tablets using the following regime: 3 × 1 mg daily for 5 days, starting at noon five days prior to the injection of the tracer, continued by 2 × 1 mg for another 7 days from the day of [^131^I]6ß-iodomethyl-19-norcholesterol injection until the day of imaging. Antihypertensive medication was continued in an unchanged manner. Spironolactone and eplerenone were not allowed to be started or reinstituted until ICAS imaging had been completed.

Patients were pre-treated with Lugol’s solution for 3 days before [^131^I]6ß-iodomethyl-19-norcholesterol injection to block unintentional free radioactive iodine uptake by the thyroid gland.

### 2.3. Acquisition Protocol

A total of 37 MBq [^131^I]6ß-iodomethyl-19-norcholesterol (Curium, Paris, France) was injected intravenously into the right cubital vein of the patients through a pre-inserted cannula. At day 7 after the injection, anterior and posterior abdominal static planar images were obtained with the patient positioned supine and the camera centered at the level of the twelfth thoracic vertebra, using an AnyScan Trio (Mediso, Budapest, Hungary) hybrid SPECT/CT device equipped with high-energy general-purpose (HEGP) collimators.

SPECT/CT images were taken with the following parameters: 120 views, 30 s/frame, continuous mode. For attenuation correction, low-dose CT was performed as follows: 120 kVp, 50 mAs, 1 pitch, 1 s rotation time, and 2.5 mm slice thickness. The duration of SPECT/CT imaging was approximately 20 min. 

3D ordered subsets expectation maximization (OSEM) reconstruction with resolution recovery (TeraTomo 3D SPECT, Mediso, Budapest, Hungary) was applied with and without attenuation correction. 

### 2.4. Image Processing

ICAS images were initially evaluated for unilateral or bilateral aldosterone secretion by two nuclear medicine specialists. The feature considered was the visual side difference, overwhelmingly suggesting one-sided adrenal tracer uptake. On the unilateral-uncertain-bilateral scale, two concordant unilateral ratings were required to declare highly probable unilateral disease. If the opinions of the two experts were discordant, a third investigator also carried out the analysis. The decision on referring to unilateral laparoscopic adrenalectomy was made solely based on the visual ICAS image-related probability of unilateral disease; uptake calculations shown below were not considered, and the investigators were not aware of the calculation results during image evaluation.

During image processing, identical spherical volumes of interest (VOIs) centered on each adrenal gland’s maximal uptake and on the liver were measured. Care was taken to include all [^131^I]6ß-iodomethyl-19-norcholesterol concentrating tissues in the adrenal region. In order to identify an optimal, universally applicable sphere size, sphere diameters between 2 and 5 cm with 5 mm increments were tested. Both mean and maximum uptake values were recorded. Ratios of the uptake of the two adrenal glands (Lateralization Index, LI) and adrenal gland/liver ratios were calculated.

### 2.5. Outcome Measures

At 6 months after surgery, the potassium level in the reference range, combined with an ARR ≤ 30, was considered as a biochemical cure [13]. The reduction in the number of antihypertensive agents taken by the patient was also recorded.

Histological assessment of the removed adrenal gland was performed in each case.

### 2.6. Statistical Analysis

The IBM SPSS Statistics version 29 software package (IBM SPSS Inc., Armonk, NY, USA) was used for data analysis. The normality of distributions was tested using the Shapiro–Wilk test. The Student’s *t*-test was used to compare parameters with a normal distribution, while the non-parametric Mann–Whitney test was applied for the comparison of variables with a non-Gaussian distribution. To identify influencing factors (ratio of the counts of the adrenal glands, adrenal gland/liver ratio, age of participants), logistic regression analysis was applied, while in ROC analysis, the maximum Kolmogorov–Smirnov metric was used to determine the optimal cut-off values for the maximum and mean tracer uptakes. *p* values below 0.05 were considered to be significant.

## 3. Results

Forty-two participants, aged 49.6 years (SD 11.1; 22 males and 20 females), were included in the analysis. The median ARR was 90.2 (Q1 = 44.2, Q3 = 148.2).

**Figure 1:**
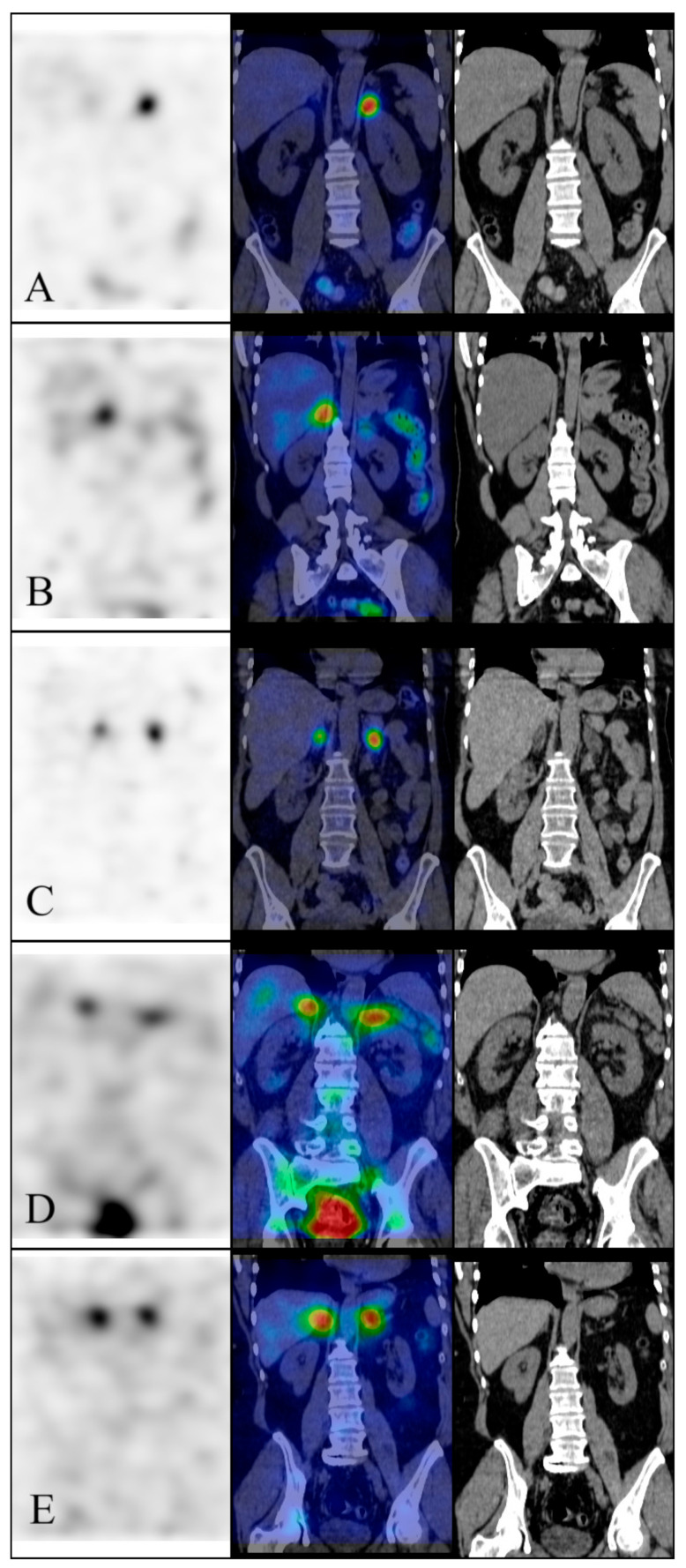
SPECT/CT images. (**A**): Unequivocal one-sided mineralocorticoid overproduction; (**B**–**D**): Cases with some indistinctness, where lateralization index calculation really helps; (**E**): Unequivocal two-sided mineralocorticoid overproduction. Lateralization indices: (**A**) 3.44, (**B**) 2.17, (**C**) 1.21, (**D**) 1.17, (**E**) 1.01. For each patient, SPECT (left), SPECT/CT fusion (middle) and CT (right) images are shown. Please see text for details of the technique.

**Figure 2:**
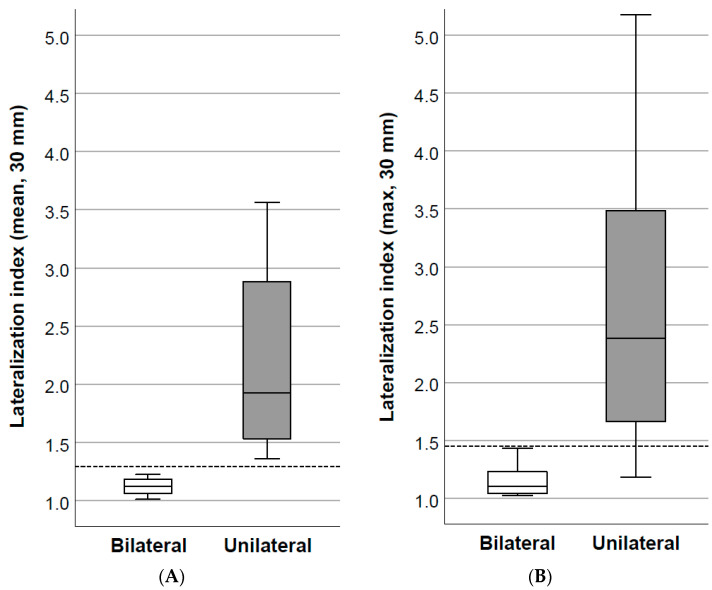
Lateralization indices calculated using mean counts (**A**) and maximum counts (**B**) of 3-cm-diameter spherical VOIs. Unilateral group *n* = 24, bilateral group *n* = 18. Box and whisker plots indicate the medians, upper and lower quartiles, and ranges.

Based on the visual evaluation of the ICAS images, 24/42 patients underwent unilateral laparoscopic adrenalectomy because of a high probability of unilateral disease (Figure 1). Of them, all 24 (100%) achieved a biochemical cure defined as a normalized potassium level combined with an ARR ≤ 30 six months after surgery. In this group, the lowest ICAS lateralization index was 1.36 (median 1.92, Q1 = 1.51, Q3 = 2.91) using mean uptakes of 3-cm-diameter spherical VOIs for calculation. A bilateral uptake by ICAS, based on the visual evaluation of the ICAS images, was found in 18 patients (Figure 1). None of the patients deemed to have bilateral disease by image inspection had a lateralization index > 1.23 (median 1.12, Q1 = 1.05, Q3 = 1.18). The lateralization indices of the unilateral and bilateral groups are shown in Figure 2.

Maximum count rates and mean count rates of the 3 cm spherical VOIs proved to be similarly effective in the separation of unilateral and bilateral disease (logistic regression, *p* = 0.00029 and 0.00031, respectively). Using the maximum Kolmogorov–Smirnov metric, the cut-off value providing the best separation of the two patient groups was found to be 1.45 and 1.29 for the maximum and mean uptakes, respectively.

Of the 24 patients with ICAS-based unilateral disease, 23 had ipsilateral adenoma by CT. One patient in this group had no adenoma by either CT or histology; histology identified hyperplasia. However, the patient achieved a biochemical cure after unilateral adrenalectomy.

Of the 18 patients with ICAS-based bilateral disease, 2 patients had unilateral adenoma on CT scan. Against our recommendation and in spite of LIs of 1.01 and 1.20, both patients underwent unilateral adrenalectomy of the CT-identified adenoma side without a resulting cure at the 6-months follow-up, the adenoma being clearly non-hormone-secreting and unrelated to the high aldosterone level.

Belonging to the unilateral or bilateral ICAS groups showed no association with the age or gender of the patients or the initial potassium level, or with the presence of adrenal abnormality revealed by CT. The mean ARR was not different in the unilateral and bilateral ICAS groups, 90.2 (Q1: 43.1, Q3: 154.3) and 97.1 (Q1: 62.2, Q3: 124.5), respectively (*p* = 0.61, ns). Adrenal-to-liver count ratios calculated using the ICAS-dominant adrenal uptake did not differentiate between unilateral and bilateral aldosterone secretion.

## 4. Discussion

Currently, less than 3% of those who would be eligible for screening towards PA are screened [14]. With a growing number of patents being identified, more will require PA lateralization. Although due to improved genetic testing and individual factors, AVS might be bypassed in a growing number of patients [15], the demand will increase for AVS or its alternatives.

For decades, AVS has been the single reliable technique for subtype identification in PA. The distinction between unilateral and bilateral disease remains crucial, as removal of an aldosterone-producing adenoma usually terminates aldosteronism and hypokalaemia and improves or cures hypertension [4,16]. Early studies with functional imaging as an alternative to AVS reported four cases using [^131^I]6ß-iodomethyl-19-norcholesterol [17], and approaches were published using [^11^C]metomidate [18] and [^68^Ga]pentixafor [19]. Recently, a large study has found [^11^C]metomidate PET/CT to be non-inferior to AVS in unilateral disease identification [20].

Cholesterol is the precursor to all steroid hormones, including cortisol and aldosterone. It takes 5–7 days for the adrenal to turn cholesterol into aldosterone. To suppress the background caused by continuous bilateral normal cortisol production, orally administered dexamethasone was used. As cortisol and aldosterone secretions of the healthy adrenal tissues were suspended in the presence of suppressed ACTH and endogenously low renin, respectively, only autonomously functioning adrenal tissue was visualized by ICAS.

For side difference evaluation, in the present study visual intensity estimation [21] was followed by the more objective LI calculation [22]. In addition to right and left maximum counts [22], we measured total counts and calculated the mean tracer uptakes of the right and left spherical VOIs. As maximum uptake calculation is device- and software-dependent, we have chosen the ratio of mean uptake values for LI calculation; it can be universally applied without the need for setting up cut-off values for each nuclear medicine unit. Our technical approach is both simple and objective, which may account for the excellent separation of one-sided and two-sided disease without overlap. A ratio ≥1.29 compared to the contralateral side was found to be diagnostic of unilateral, renin-independent aldosterone secretion. This discrimination criterion also worked in the three unusual cases in our series: in two patients with bilateral disease by ICAS, CT scan found an unequivocal nodule in one of the adrenals. Indeed, removal of the nodule-containing adrenal failed to result in biochemical recovery. A similar phenomenon was described by others [23,24]. In another patient, both adrenals were intact by CT, but ICAS revealed a clear side difference; biochemical recovery resulted after unilateral adrenalectomy. This already described constellation [25,26] is called ‘lateralized hyperaldosteronism’ [4]. To complicate all this further, due to the molecular heterogeneity of adrenal micronodules [23], theoretically unilateral micronodules may be the source of PA even in the presence of a non-secreting ipsilateral larger adenoma detected by CT. Thus, a new advantage of functional imaging emerges, i.e., certain cases that are currently not sent to AVS due to the absence of an adenoma on CT may have a side difference by ICAS, which does initiate the consideration of surgery of the involved side. All the above point to the superiority of functional imaging over AVS.

To evaluate the accuracy of ICAS-driven decisions towards unilateral adrenalectomy, outcome measures defined by an international group of investigators were used [13]. We considered only complete biochemical success; we believe that the lack of testing for partial biochemical success might not have substantially distorted the results, as the partial success rate was 4% in an international study [13]. In the present series, biochemical cure after ICAS-based adrenalectomy was 100%. This diagnostic accuracy is comparable to that reported for AVS [27]. The results by three groups using the same radionuclide were close to our findings [21,22,24]. In one of these series [22], AVS was used as the reference, while the other two, similarly to us, used the surgical outcome [21]. We believe that the latter approach is closer to real life; both AVS and ICAS have their own inherent weaknesses, which may equally account for the not very strong correlation between them. Using ICAS, we found a clear separation of unilateral and bilateral disease, with no overlap between the two groups. This excellent discrimination can be attributed to the patient preparation and the new image evaluation technique we used. However, a continuum instead of dichotomy of side differences with ICAS may be expected in larger studies, because the number of adrenal micronodules, which are not fully symmetric, increases with age [28]. Interestingly, our findings are nearly identical with those of the very first human study with ^131^I-labeled cholesterol nearly half a century ago [29].

One potential drawback of the ICAS technique is the 12-day oral dexamethasone suppression, which may have side effects. In the present series, serum potassium remained in the pre-test range. We speculate that lack of further decrease in the potassium level while on dexamethasone may be explained by the already full stimulation of the mineralocorticoid receptors by the high aldosterone level and that dexamethasone cannot add to this. Based on our experience, no additional serum potassium level measurements are required while the patient is on dexamethasone, provided that the previous therapeutic regime is continued.

A clear advantage of ICAS over AVS is that it does circumvent several disease-heterogeneity-related weaknesses of AVS. Theoretically, one-sided overproduction of any adrenal steroid, including both aldosterone and 11-deoxy-corticosterone (DOC), can be detected by ICAS. The co-secretion of aldosterone and cortisol is also expected to provide an elevated, and thus diagnostic, LI by ICAS; this co-secretion usually results in a misleadingly low aldosterone-to-cortisol ratio by AVS at the involved side [30]. Further, AVS cases in which adrenal vein catheterization has failed in one of the two sides are usually not included in published surgical outcome series, hindering real comparisons. As with AVS, adrenal cancer has to be ruled out by conventional imaging. Cost comparisons also favor ICAS over AVS; [^131^I]6ß-iodomethyl-19-norcholesterol is widely available, and the technique can be easily performed by any nuclear medicine unit without a long learning curve. Shared decision making with the patient and an interdisciplinary approach remain a must.

The calculated radiation exposure of ICAS is 22 mSv (20 mSv radionuclide + 2 mSv low-dose CT). In comparison, during AVS, the reported exposure ranges from 3.2 to 29 mSv [31]. The effective dose of a multiphase abdominal CT is approximately 25 mSv.

A limitation of the present study is that a comparison with AVS was not performed. However, the outcome-verified approach, i.e., post-surgery biochemical success in one-sided ICAS-positive patients (those with an LI ≥ 1.29), argues for the value of the technique. We are aware of the fact that some of the two-sided patients by ICAS, who were *per protocol* not operated on, might have had one-sided disease; however, this may not be frequent, as the prevalence of outcome-verified one-sided disease was close to the reported one [11]. A rational approach is to not refer to surgery those patients who do not have an LI ≥ 1.29, irrespective of the presence or absence of adrenal adenoma on cross-sectional CT images.

Why the studies published over the last decade with [^131^I]6ß-iodomethyl-19-norcholesterol did not result in the replacement of the invasive, and in 15% of the cases, inconclusive AVS is not clear. One novelty described in the present study is the VOI-mean-count-based image interpretation and side ratio calculation, instead of the visual-scale-based side-difference estimation. Our technique is manufacturer- and software-independent, i.e., may be applied to any SPECT/CT unit. All this may facilitate a more widespread use of the technique. 

[^131^I]6ß-iodomethyl-19-norcholesterol SPECT/CT reliably identifies a unilateral adrenal aldosterone source and facilitates patients’ selection for surgical intervention in primary aldosteronism. This functional imaging may replace AVS if lateralization is required for management decisions in primary aldosteronism.

## Data Availability

The data presented in this study are available on reasonable request from the corresponding author. The data are not publicly available due to the presence of patient identifiers on images.
